# Contagious Cerebro-spinal Meningitis

**Published:** 1898-09

**Authors:** 


CONTAGIOUS CEREBRO-SPINAL MENINGITIS. Recently a virulent outbreak occurred in a barn containing forty head of draught-horses, and of which number three had already died, was effectually checked by thoroughly washing the interior of the building with boiling water containing two ounces of carbolic acid crystals to each gallon. None of the remaining horses was affected after this method of disinfection had been employed.

